# Thyroid Nodule Imaging, Status and Limitations

**Published:** 2015

**Authors:** Durr-e-Sabih, Kashif Rahim

**Affiliations:** Multan Institute of Nuclear Medicine and Radiotherapy, Multan, Pakistan

## Thyroid nodules

Thyroid nodules are common, occurring in almost two-thirds of some populations; among these only about 7% are malignant (1). The most important question with any new discovered thyroid nodule is, “is this malignant?” The main arbiter of malignancy or benignity remains fine needle aspiration. Given the resources involved, doing a fine needle aspiration cytology (FNAC) in every discovered nodule would be prohibitive to impossible. The clinician must decide which nodule to investigate and which to watch in the hope that this will never turn out to be malignant. FNACs are used basically to decide which nodule to operate upon (or more importantly which to not operate upon) and clinical and imaging features are used to decide which nodule to investigate by FNAC and which to leave alone. This paper describes the various imaging options for looking at thyroid nodules and briefly discusses the advantages and disadvantages with each.

### Clinical

Features that raise suspicion include a nodule in males, one that is solitary, growing, hard, fixed and associated with hoarseness (2). While the presence of these features is likely to be of concern for a thyroid cancer, only very few patients of thyroid malignancy present in this classical manner. Several studies have shown women to outnumber men in a thyroid cancer population, cancers in Multinodular Goiters (MNG) to be far more common than in solitary nodules (3-5) and small impalpable nodules as likely to be malignant as larger more obvious ones (6). This means additional information is needed if some patients are to be stratified into a “low-risk, observe-only” group. Various imaging modalities are available that allow, with varying degrees of success and accuracy, the classification of nodules in to benign and malignant groups.

### Nuclear Medicine

**Figure 1:**
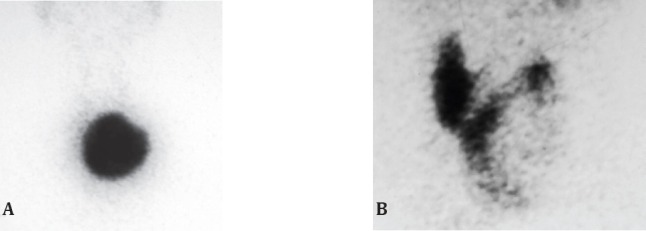
^99m^TcO4 thyroid scan showing, A; a large hot nodule with suppression of the rest of the thyroid and B; a large cold nodule in the left lobe of the thyroid. On cytology the hot nodule was reported as a “follicular neoplasm” and the cold nodule was reported as a “colloid nodule”

Thyroid imaging with radioactive iodine and Technetium (^99m^Tc) was the monopoly of nuclear medicine for over a quarter of a century with hot nodules interpreted to be benign and cold possibly malignant, Figure 1. As more specific imaging developed, the utility of Iodine and Technetium scanning declined so that it is no longer a preferred investigation when a thyroid nodule is first diagnosed. Radionuclide thyroid scans do not differentiate benign from malignant (7), do not alter therapy and do not come cheap (8). Hot nodules have a reported incidence of malignancy (9, 10). The only situation where a hot nodule can be placed in a low risk category is when there is suppressed Thyroid Stimulating Hormone (TSH) level (11). Other nuclear medicine were developed to lend specificity to the images, these include Thallium, sestamibi, tetrorosminetc but although useful in niche situations, did not demonstrate sufficient sensitivity and specificity (12, 13) to find acceptance in routine initial imaging of a thyroid nodule.

### Positron Emission Tomography (PET)

**Figure 2:**
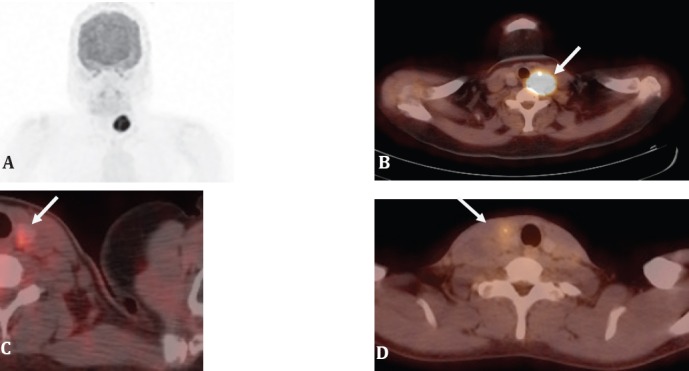
Spectrum of metabolic activity of thyroid carcinoma on PET imaging. A, B; Left lobe papillary thyroid carcinoma showing increased glucose metabolism with a SUV_max_ of 17.9. C; Left lobe papillary carcinoma with relatively low level of glucose metabolism, SUV_max_ 3. &. D; right lobe papillary carcinoma with a low glucose metabolism with a SUV_max_ of 2.7. (*Images A, B and D courtesy Prof. Dr. Jun Hatazawa. Osaka University Graduate School of Medicine, Japan; Image C, courtesy Prof. Dr. Henry Bom, Hwasun, Korea*)

**Figure 3:**
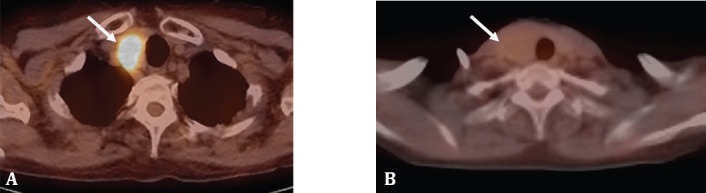
Spectrum of metabolic activity in benign thyroid nodular disease on PET. A; Right lobe benign follicular adenoma showing increased glucose metabolism on PET with an SUV_max_ 10.5. B; Right lobe follicular adenoma showing very subtle, low metabolic activity with and SUV_max_ of 3.1 (*Image A, Courtesy Prof Henry Bom. Hwasun Korea. Image B, Courtesy Prof. Dr. Jun Hatazawa, MD, PhD Osaka University Graduate School of Medicine*)

The practice of oncology has changed with the introduction of PET scanning, and PET is used in the initial diagnosis, staging, follow-up, re-staging and prognostic assessment in many cancers. Well differentiated thyroid cancers (WDTC) unfortunately do not lend themselves to an initial PET assessment due to wide variability of results (14) (Figure 2, 3). There is however an indispensable place for PET imaging in treated Thyroglobulin (TG) positive Whole-body Iodine Scan Negative cases (15).

### Computerized Tomography and Magnetic Resonance Imaging

**Figure 4:**
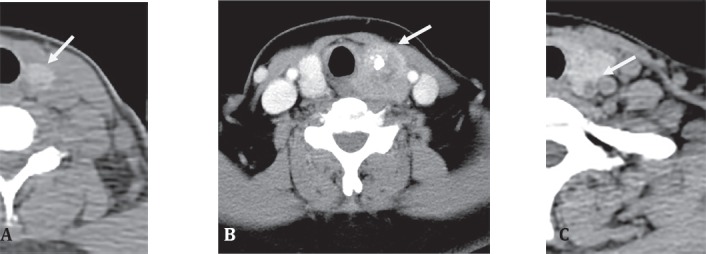
CT scans of thyroid nodular disease. A, B, Papillary carcinoma showing a hyperattenuating mass in the left lobe of the thyroid and a complex mass with calcification in the left lobe of the thyroid (*Image courtesy Prof. Dr. Jun Hatazawa, Osaka University Graduate School of Medicine*) c, benign adenoma seen as a complex mass deep within the left lobe of thyroid (*Image courtesy Prof. Dr. Henry Bom, Hwasun, Korea*)

The level of anatomical detail in computerized tomography (CT) and MR have made these the investigation of choice for most macroscopic pathological processes Figure 4 and there is a high number of thyroid incidentaloma discovery on CT/MR of the neck done for other indications. Despite the resolution that is achievable, initial thyroid nodule imaging with CT/MRI has mixed reviews with some suggesting that all such nodules be assessed by ultrasound (16) while others find these modalities useful in giving added specificity and confidence in stratifying patients who can avoid FNAC without missing significant disease (17).

### Ultrasound

A consensus that has emerged over the last few years is that ultrasound currently offers the most valuable tool in the early assessment of thyroid cancer (18). The advantages of ultrasound examination of thyroid include:

- The ability to identify non-palpable nodules
- Accurately measure nodule and detect any interval change in size
- Differentiate thyroid from non-thyroid nodules (lymph nodes, thyroglossal cyst, cystic hygroma, vascular malformations etc) (19, 20)
- Identify cervical lymphadenopathy and characterize enlarged nodes into benign and malignant (21)
- Stratify thyroid nodules according to probability of malignancy (22-24)
- In MNG select nodules for FNAC
- Evaluate residual thyroid tissue after surgery
- Evaluate diffuse thyroid changes (25)
- Guide needle tip placement for FNAC

**Figure 5:**
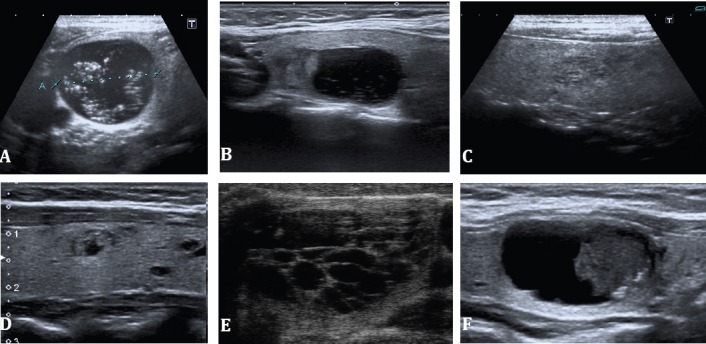
Spectrum of findings in benign thyroid nodules. A, B; colloid cysts, the Echogenic specs in both purely cystic lesions are colloid crystals. C, D;microcystic or spongiform nodules, these are benign colloid nodules. E, F; hemorrhagic cysts showing a reticular appearance in E, and retracted clot in F

**Figure 6:**
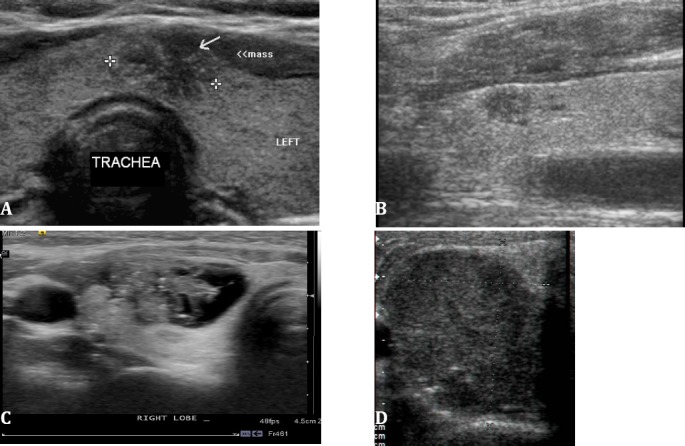
Spectrum of findings in malignant thyroid nodules (all cases of papillary carcinoma). A, small isthmic mass with an irregular contour, marked hypoechogenicity and microcalcifications. B, marked hypoechogenicity, pointed margins in the upper pole of the right lobe. C, complex mixed cystic and solid mass with contour irregularity laterally and microcalcifications. D, uniformly solid, markedly hypoechoic taller than wide mass with a few internal microcalcifications. (Image A courtesy Dr. Ravi Kadasne, UAE. Images B-,D courtesy Shlomo Gobi, Jerusalem)

Given the ubiquity of thyroid nodules, it would be impossible to biopsy all nodules for selection for surgery and ultrasound criteria have been used to try and divide nodules into those that are suspicious or look malignant and those in whom biopsy can be deferred (Figure 5, 6). All ultrasound modalities including gray-scale, Doppler, elastography as well as contrast have been used to differentiate benign from malignant nodules. Gray scale and Doppler characteristics have been well defined that help in categorization of the nodules.

Several benign and malignant ultrasound gray scale and Doppler features have emerged over the last few years (22, 26) (Table 1). These can be used in different ways to assign probabilities and a method based on the Breast Imaging Reporting and Data System (BIRADS) system, called Thyroid Imaging Reporting and Data System (TIRADS) was described in 2009 (24, 27) (Table 2).

**Table 1:** Ultrasound features associated with Benign or Malignant Probability of Thyroid Nodules

Benign	Malignant
Uniform Halo	Microcalcification
Predominantly Cystic	Extension beyond thyroid
Avascular	Metastatic nodes
Reverberating echogenicities	Taller than Wide
	Hypoechoic
	Irregular Margin
	Solid
	Increased Central Vascularity

**Table 2:** Ultrasound features of different classes of TIRADS system (24)

Group	Significance, (%probability of malignancy)	US Pattern	Comment
TIRADS 1			Normal
TIRADS 2	Benign (0%)	Colloid 1	Anechoic, avascular, echogenic spots
		Colloid 2	Nonencapsulated, mixed, non expansile, hyperechognic spots, vascularized, spongiform
TIRADS 3	Probably benign (<5%)	Colloid 3	Non-encapsulated, solid/cystic, iso/hyperecohgenic, expansile, vascualzed, hyperechoic spots
		Hashimoto pseudonodule	Hyper/iso/hypoechogenic, partially encapsulated, peripheral vascularity in background of Hashimotos thyroiditis
TIRADS 4	4A, Suspicious (5-10%)	Simple neoplastic	Solid or heterogeneous nodule with thin capsule
		De Quervain pattern	Hypoechoic ill defined lesion, without calcification
	4B, Suspicious (10-80%)	Suspicious neoplastic	Hyper/iso/hypoechoic, hypervascularized, thick capsule, calcification
TIRADS 5	Probably Malignant (>80%)	Malignant B	Iso/hypoechoic, nonencapsulated, multiple peripheral calcifications and increased vascularity
		Malignant C	Malignant A, without calcification
TIRADS 6	Biopsy Proven		

There are several methods of characterization of ultrasound features. The original TIRADS papers (24, 27) describe an extensive spectrum of benign and malignant features. Despite their comprehensiveness, or perhaps because of it, the original TIRADS schemes are difficult to apply and have a very long learning curve. The description of nodules is complex and in real-life one is often unsure which category to put a particular nodule in.

There has been extensive work on determining the statistical significance of each ultrasound feature in predicting malignancy (7, 11, 22, 23). Using the established features of malignancy, simplified, easier to use methods of thyroid nodule classifications have been proposed (22, 23). These schemes assign probability of malignancy depending upon how many suspicious features are present in a nodule. Some schemes assign weighting factors to pre-existing high risk conditions (Table 3). Most malignant nodules are seen to have at least two suspicious features, but nodules with even one suspicious feature are a candidate for biopsy (22).

**Table 3:** American Thyroid Association Criteria for biopsy of a Thyroid Nodule (11)

	US feature	Threshold
High risk history of Thyroid Cancer in first degree relatives, history of childhood radiation to neck, previous cancer in contralateral lobe. FDG avidity	Solid, suspicious features: Microcalcification, hypoechoic, irregular, taller than wide on transverse view	> 5 mm
	No suspicious features	0.5-1.5 cm
	Abnormal nodes	All
	Microcalcification	All
Solid Nodule	Hypoechoic	>1 cm
	Hyperechoic	>1.5 cm
Mixed solid-cystic	With suspicious features	1.5-2.0 cm
	Without suspicious features	> 2.0 cm
Spongiform		Not indicated but FNAC node if present
Purely cystic		Not indicated

Sensitivities and specificity for the various schemes have been worked out (28). Sensitivity ranges from 60% to nearly 94% (Table 4).

**Table 4:** Performance of various classification schemes for thyroid nodules. From ref (28)

Study	Diagnostic Criteria	Sensitiviy %	Specificity %	Ppv	Npv	Accuracy
TIRADS (Kwak et al.(22))	Benign: TIRADS 2, 3, 4a Malignant: TIRADS 4b, 4c,5	60.2	85	75.5	73.6	74.2
TIRADS (Horvath (24))	Benign: TIRADS 1, 2, 3 Non-benign: TIRADS 4, 5	88	49	49	88	94
Diagnostic categories (Kim(43))	Sonographic characteristics: Microcalcification, Irregular or lobulated margins, marked hypoechogenicity and more tall than wide	93.8	66	56.1	95.9	74.8

The later schemes, especially of Kwak, are simple enough to be practically implementable by even less experienced radiologists (29, 30).

A recently published retrospective study with 8806 patients (31) used only three ultrasound characteristics, microcalcifications, size >2 cm and an entirely solid composition. By using two characteristics, the authors claim that 90% biopsies would be avoided with a residual thyroid cancer rate of 5 per 1000 with sensitivity of 0.52 and false positive rate of 0.07. The same issue of the journal carries a cautionary comment (32) on implementing this rather simplistic approach and recommends biopsy for the nodules larger than 1 to 1.5 cm, solid and hypoechoic with microcalcifications.

Work has been done on trying to identify, with certainty, benign nodules on ultrasound (33-35) and criteria for benign nodules are emerging too. Tay SY et al. (33) concluded that the nodules with well-defined margins, no calcification, normal vascularity and negative lymphadenopathy should allow follow up. Vinyak S. et al. (34) described that the nodules with regular margins, having homogenous texture, normal vascularity and no calcification need follow up without FNAC. Brito et al. (35) reported that pure cystic or spongiform nodules do not need FNAC and require follow up.

## Discussion

The management of thyroid nodules has become increasingly complex. In almost all other cases of focal disease, the aim of early detection is to identify cases that might be malignant, so that an appropriate intervention can be planned. Managing cases of early thyroid cancer is not so straight forward. Most patients of thyroid cancer will die with thyroid cancer but not of thyroid cancer (32). Tools for early diagnosis, especially ultrasound, are picking up smaller and smaller thyroid cancers, resulting in an epidemic of sorts of thyroid cancers (36, 37). The number of new cases of thyroid cancer have tripled in recent years from 4.8 per 100,000 in 1972 to 14.7 per 100,000 in 2011, cancer mortality has remained stable at about 0.5 per 100,000 during this period (38). Some autopsy series have picked up undetected thyroid cancers in up to a third of all cases (39). Very small papillary thyroid carcinomas are already being classified into microcarcinomas and even smaller latent microcarcinomas that are less than 1-3 mm across (40). With the strides that ultrasound resolution is making it is already possible to identify and characterize thyroid nodules that are 2-3 mm in size. This barrier is likely to be temporary and soon we should expect millimeter resolution for ultrasound detection thresholds.

This raises two important questions in patients with palpable or impalpable thyroid nodules; is this likely to be malignant? And, if malignant, does this warrant aggressive treatment? There are no easy answers to these questions, especially the second, but due to resource constraints some stratification strategy that will obviate biopsy as a first step is needed.

The whole discussion of thyroid imaging boils down to establishing thresholds at which to decide if biopsy needs to be done or not. The American Thyroid Association has laid down very detailed criteria of biopsy thresholds (11). These criteria are dynamic, and take into account not only the imaging characteristics of the thyroid nodules but also the clinical and individual context of the patient so that the thresholds are lower for a patient with a high risk background (Table 3). There are other biopsy criteria too, each having advantages and trade-offs (41).

Many groups have demonstrated sufficient confidence in their ultrasound findings to use the FNAC results only to confirm their initial ultrasound impression. In cases of discordant FNACs (malignant ultrasound features, benign FNAC results), they suggest a second FNAC reading (42).

Ultrasound imaging has been used, though with less-than-perfect sensitivity, for identifying patients who do not need an FNACC but more work is needed to ensure that the patients who are not biopsied do not develop cancer at a later stage. The development of new applications and new methods of extracting information from ultrasound data like elastography and contrast will bring in more sensitivity and specificity to the diagnostic process. At present we can confidently state that ultrasound imaging offers a powerful tool to help in making management decisions in thyroid nodular disease.
